# Is contrast-enhanced endoscopic ultrasound-guided fine needle biopsy better than conventional fine needle biopsy? A retrospective study in a medical center

**DOI:** 10.1007/s00464-022-09253-3

**Published:** 2022-04-28

**Authors:** Jian-Han Lai, Ching-Chung Lin, Hsiang-Hung Lin, Ming-Jen Chen

**Affiliations:** 1 Division of Gastroenterology, Department of Internal Medicine, Mackay Memorial Hospital, No. 92, Sec. 2, Chung-Shan North Road, Taipei, Taiwan; 2 Mackay Medicine, Nursing and Management College, Taipei, Taiwan; 3 Mackay Medical College, New Taipei, Taiwan

**Keywords:** Contrast, Endoscopic ultrasound, Fine needle biopsy, Pancreas

## Abstract

**Background:**

Contrast-enhanced endoscopic ultrasound-guided fine needle aspiration (CE-EUS-FNA) could help clinicians to precisely locate and puncture lesions, but its effect on the diagnostic yield improvement is controversial. We designed this study to observe the additional benefit of using contrast in EUS-guided tissue sampling while performing fine needle biopsy (FNB) instead of FNA, as FNB results in a higher diagnostic accuracy.

**Method:**

Patients who underwent EUS-FNB performed by a single medical team from January 2019 to March 2021 were included in this study. We analyzed the cytopathological diagnostic accuracy rate and number of needle passes between groups who underwent FNB with and without contrast.

**Result:**

We divided 133 patients who were diagnosed with a malignancy into two groups according to whether they underwent CE-EUS-FNB (*n* = 48) or conventional EUS-FNB (*n* = 85). The CE-EUS-FNB group had an equal diagnostic accuracy rate with fewer needle passes compared with the conventional EUS-FNB group. There was no significant trend change in the success cytopathological diagnostic rate for experienced endoscopists for EUS-FNA.

**Conclusion:**

CE-EUS-FNB had fewer needle passes but no additional benefit for diagnostic yield improvement. There was no difficult threshold for CE-EUS-FNB for endoscopists who were well trained in conventional FNA.

Endoscopic ultrasound (EUS) is a useful method for surveying pancreatobiliary lesions. Furthermore, contrast-enhanced EUS (CE-EUS) can provide more information to diagnose lesions [1]. Fine needle aspiration or biopsy (FNA or FNB) can then be used for the final cytopathological diagnosis. Contrast-enhanced harmonic EUS-guided FNA (CE-EUS-FNA) could help us to precisely focus the puncture target [2]; however, the diagnostic yield over other methods is controversial [3–5]. Thus, we designed this study to observe the additional benefits of CE-EUS-guided tissue sampling. We used an FNB needle instead of an FNA needle because the former can provide a higher diagnostic accuracy [6].

## Materials and methods

### Patients

The Institutional Review Board of Mackay Memorial Hospital, Taipei, Taiwan approved the protocol for this retrospective study. We reviewed the cases of patients who underwent EUS-FNB for pancreatic tumors or retroperitoneal lymph nodes at Mackay Memorial Hospital between January 2019 and March 2021. Personal and clinical data, including age, sex, chronic pancreatitis presentation, contrast used during EUS-FNB, tumor location, tumor size, number of needle passes, FNB cytopathological results, and final diagnosis, were extracted from the patient records for analysis.

An unsuccessful FNB cytopathological diagnosis was defined as either a false negative or atypical result, while a successful FNB diagnosis was defined as a suspicious or positive finding of malignancy. If a patient had an unsuccessful FNB cytopathological diagnosis, we arranged for further surgical direct biopsy or transabdominal echo-guided metastatic lesion biopsy to obtain the final histological diagnosis. Patients who were diagnosed with benign lesions underwent diagnostic imaging follow-up for at least 6 months to rule out the possibility of a missed diagnosis of malignancy. We excluded patients in whom the final diagnosis was uncertain.

### Procedure

All EUS-FNB procedures were performed by two endoscopists who achieved the FNA learning curve. The EUS procedures were performed using a curvilinear echoendoscope (GF-UCT260, Olympus, Japan), and aspiration was performed using a 22-gauge FNB needle (Acquire™, Boston Scientific, USA). We only used contrast (Sonazoid, GE Healthcare, USA) in patients who agreed to self-pay because it was not covered by health insurance in our country. The standard dose of contrast was 0.015 mg/kg and then FNB was performed under CE guidance into the hypo-enhanced area of the tumor [2]. Other patients who opted to not self-pay for the contrast underwent the conventional FNB procedure. A fanning method was used for FNB, with aspiration from at least four different areas within the target lesion using a stylet slow-pull or low-negative suction technique. The endoscopists then fixed the acquired tissues in ethanol and formalin for preparation as cytological smears and pathological samples, respectively. Individually, both endoscopists decided on the number of FNB passes required for each case based on the volume of the obtained tissue (macroscopic on-site quality evaluation) [7]. Rapid on-site cytological evaluation was not available in our hospital setting.

### Statistical analysis

Continuous variables are reported as mean ± standard deviation, and categorical variables are reported as frequencies and percentages. Independent sample *t* test, Chi-square test, and crosstabs statistics were used, according to the data type, to compare the baseline clinical characteristics between the CE and conventional EUS-FNB diagnostic groups. All analyses were performed using the SPSS 21.0 statistical package (SPSS, Chicago, IL, USA), with a two-sided *p*-value of 0.05 considered as significant.

## Results

A total of 155 patients underwent EUS-FNB at our hospital during the study period. The clinical and EUS characteristics of the patients are shown in Table 1. We excluded three patients with uncertain diagnoses and who were lost to follow-up in our hospital. A total of 140 patients had successful cytopathological diagnoses from EUS-FNB (121 malignancies and 19 benign), with sensitivity, specificity, and accuracy of 91%, 100%, and 92.1%, respectively. The median number of FNB needle passes was three, ranging from one to six. Twelve patients with an unsuccessful FNB cytopathological diagnosis were diagnosed by surgical direct biopsy (*n* = 7) and transabdominal ultrasound biopsy (*n* = 5).

**Table 1:** Clinical and EUS characteristics of patients who underwent endoscopic-guided fine needle biopsy (*n* = 155)

Age, years, mean ± SD (range)	63.64 ± 12.58 (31–88)
Sex, male/female, *n*	72/83
Chronic pancreatitis, *n* (%)	20 (12.9%)
Tumor location, *n*^a^	78 (50.3%)/57 (36.8%)/20 (12.9)
Tumor size, cm, mean ± SD (range)	3.18 ± 1.60 (0.7–12)
Numbers of FNB pass, median (range)	3 (1–6)
Malignant/Benign lesion, *n*^b^	133 (87.5%)/19 (12.5%)
Adc, pNET, malignant lymph node, *n*	92 (69.2%)/18 (13.5%)/23 (17.3%)
Success cytopathological diagnosis, *n*^b^	140 (92.1%)

*SD* standard deviation

^a^Pancreas uncinate process and head/body and tail/lymph nodes

^b^Three patients had no final diagnosis

Nineteen patients with a benign diagnosis had a diagnostic imaging follow-up of more than 6 months, and no patient was diagnosed with a malignancy during the follow-up period. The diagnostic modalities used in the follow-up imaging studies included pancreatic CT scan and MRI. There were 133 patients with a confirmed malignancy, which included 92 patients with adenocarcinoma, 18 patients with neuroendocrine tumors, and 23 patients with metastatic lymph nodes. The origin of the metastatic lymph nodes included the following: lymphoma, lung adenocarcinoma, squamous cell lung carcinoma, sarcoma, hepatic cell carcinoma, hepatic cholangiocarcinoma, renal cell carcinoma, gastrointestinal stromal tumor, and myeloma.

There were no significant differences in the presentation of chronic pancreatitis, tumor size, tumor location, and operators between the patients who were successfully diagnosed (*n* = 140) and unsuccessfully diagnosed (*n* = 12) by FNB cytopathology (not shown in table). All 12 patients who were unsuccessfully diagnosed by FNB cytopathology had no chronic pancreatitis.

Further analysis was performed on the data of the 133 patients with a confirmed malignancy (Table 2). Among these patients, CE-EUS-FNB was performed in 48 patients. There were no significant differences in the presentation of chronic pancreatitis, tumor location, tumor size, and success rate of the cytopathological diagnosis between the groups with and without contrast. However, the number of needle passes was lower in the contrast group (2.21 ± 0.68) than in the conventional group (3.64 ± 1.2). This indicates that the FNB cytopathological diagnostic rate was similar in these two groups, but fewer needle passes were necessary in CE-EUS-FNB.

**Table 2:** Comparison of personal and clinical factors of 133 patients with malignancy who underwent fine needle biopsy with and without contrast

Variable	Contrast-enhanced guided (*n* = 48)	Conventional (*n* = 85)	*p*-value
CP, *n* (%)	6 (12.5%)	7 (8.2%)	0.545
Tumor location, *n*^a^	29 (60.4%)/11 (22.9%)/8 (16.7%)	39 (45.9%)/36 (42.4%)/10 (11.8%)	0.078
Tumor size, cm^b^	2.95 ± 1.15	3.48 ± 1.82	0.323
Pass number, *n*^b^	2.21 ± 0.68	3.64 ± 1.20	0.000
Cytopathological result^c^	2 (4.2%)/2 (4.2%)/44 (91.7%)	6 (7.1%)/2 (2.4%)/77 (90.6%)	0.456
Success diagnosis	44 (91.7%)	77 (90.6%)	0.835

*SD* standard deviation, *CP* chronic pancreatitis

^a^Pancreas uncinate process and head/body and tail/others

^b^Mean ± standard deviation

^c^False negative/atypia/positive for malignancy

We further analyzed the differences in tumor location, and fewer needle passes were also observed in CE-EUS-FNB than in conventional EUS-FNB in all subgroups (Table 3). The mean number of needle passes in the contrast group was 2.17, 2.45, and 2 in the uncinate process and head, body, tail, and lymph node, respectively; these were less than those of conventional EUS-FNB (3.64, 3.67, and 3.5), and there was a significant statistical difference. This indicated that CE-EUS-FNB required fewer needle passes, regardless of the puncture target location.

**Table 3:** Comparison of the cytopathological diagnostic rate and number of needle passes in different tumor locations in patients with malignancies with and without contrast

	Uncinate and head cancer (*n* = 68)				Body and tail cancer (*n* = 47)			Lymph node (*n* = 18)		
	Contrast (*n* = 29)		No contrast (*n* = 39)	*p*-value	Contrast (*n* = 11)	No contrast (*n* = 36)	*p*-value	Contrast (*n* = 8)	No contrast (*n* = 10)	*p*-value
Success cytopathological diagnosis, *n* (%)	27 (93.1%)		35 (89.7%)	1.000	11 (100%)	34 (94.4%)	1.000	6 (75%)	8 (80%)	1.000
Pass number^a^	2.17 ± 0.76 (1–4)		3.64 ± 1.04 (2–6)	0.000	2.45 ± 10.69 (1–3)	3.67 ± 1.39 (1–6)	0.018	2 ± 0 (2)	3.5 ± 1.18 (2–6)	0.009

^a^Mean ± standard deviation (range)

**Fig. 1:**
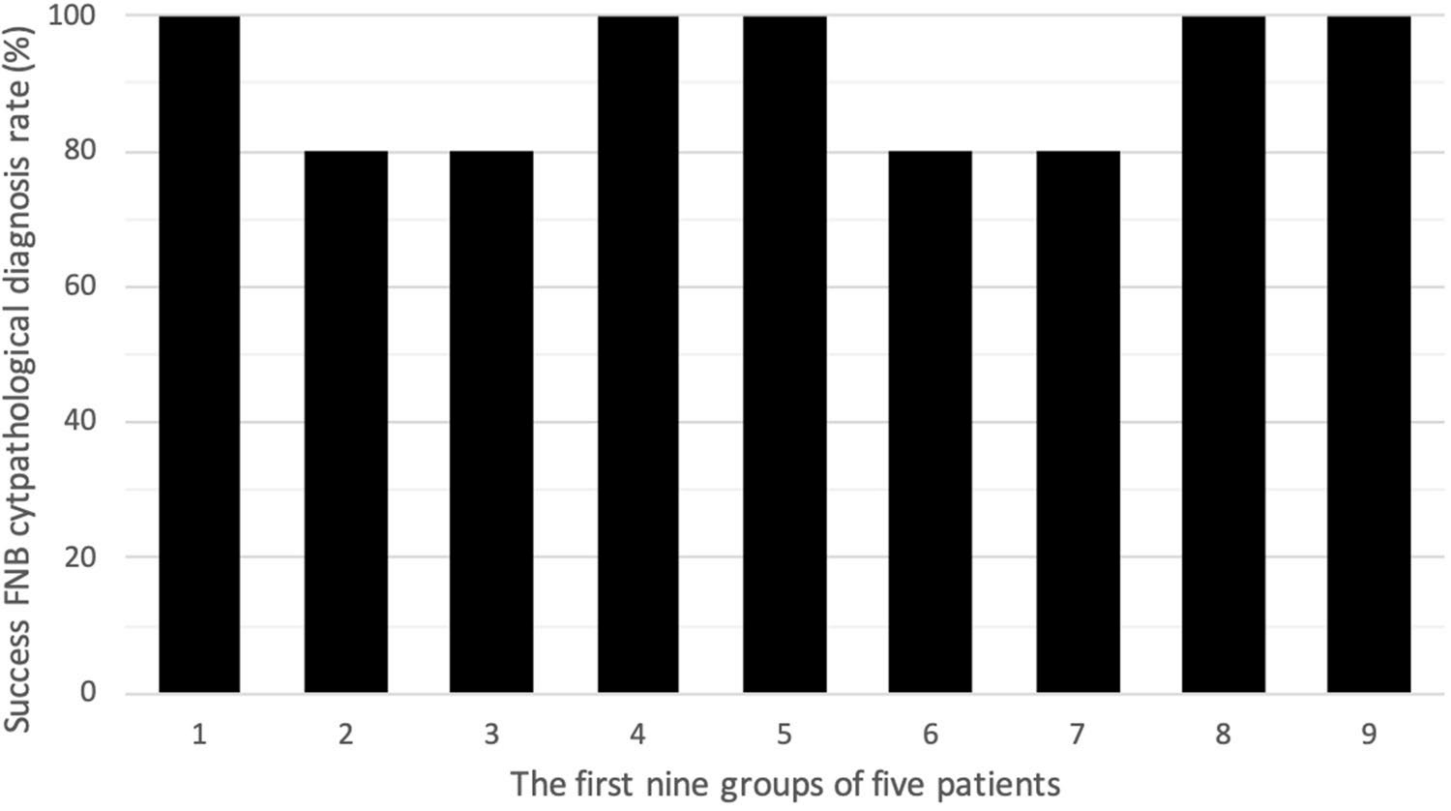
The percentage of successful cytopathological diagnosis by FNB in the first nine groups of five patients

We also divided the patients with malignancies in the contrast group into subgroups based on the time sequence and observed for changes in the rate of successful FNB cytopathological diagnosis (Fig. 1). There was no obvious difference in the success rates along the time sequence. This indicated that CE-EUS-FNB had no difficult threshold for endoscopists who were well trained in conventional FNA.

## Discussion

CE-EUS provides additional information for diagnosing pancreatic lesions [8]. The hypo-enhanced pattern in CE-EUS images had a sensitivity of 88–96% when detecting pancreatic adenocarcinomas [9]. The other classical CE-EUS features of pancreatic adenocarcinoma include a non-homogeneous network and fast washout. In contrast, pancreatic neuroendocrine tumors have a hyper-enhanced and slow washout pattern [10]. However, even with the high diagnostic accuracy of CE-EUS, the final diagnosis of pancreatic lesions should still be made based on the cytological or histological results [11, 12].

CE-EUS-FNA could identify the target area and avoid the avascular area [2]. Some studies have shown that CE-EUS-FNA had a higher adequate sampling rate and sensitivity than conventional EUS-FNA, especially for tumors > 15 mm [4, 13]. A meta-analysis study also showed that the pooled diagnostic sensitivity with CE-EUS-FNA and conventional EUS-FNA were 84.6% and 75.3%, respectively (*p* < 0.001) [5]. However, some studies have shown that there was no significant difference in the diagnostic rate between CE-EUS-FNA and conventional EUS-FNA [3, 4, 14–16]. Despite this, fewer needle passes are required to obtain abundant cytological material in CE-EUS-FNA [3, 17]. Therefore, CE-EUS-FNA may be more efficient than conventional EUS-FNA for tissue sampling of solid pancreatic lesions [17]. In our study, an equal cytopathological diagnostic rate with fewer needle passes was noted in the CE-EUS-FNB group compared with the conventional EUS-FNB group. This finding was comparable to the results of other FNA studies. Furthermore, regardless of the tumor location, tumor size, and presentation of chronic pancreatitis, CE-EUS-FNB had no additional benefit for the diagnostic yield improvement.

The benefit of fewer needle passes could shorten the procedure time. A meta-analysis based on 11,652 patients showed that fewer needle passes (< 3) could decrease the risk of post-procedural bleeding [18]. This indicates that CE-EUS-FNB could decrease the number of needle passes, and it might be helpful for avoiding bleeding complications. However, because the risk of FNA/B bleeding was determined to be very low (0.8%) [18], the real benefit of fewer needle passes to avoid complications remains uncertain, and further studies are needed.

With regard to the type of fine needle device, the FNB needle was superior to the FNA needle because it provided a higher pooled diagnostic accuracy (87% vs. 80%; *p* = 0.02) and fewer passes (*p* = 0.03) in a meta-analysis study [6]. Therefore, we used the FNB needle in the present study. In this study, the sensitivity, specificity, and accuracy of FNB cytopathological diagnosis were 91%, 100%, and 92.1%, respectively. This finding was compatible with the results of a multicenter randomized controlled trial [19]. The number of conventional FNB needle passes in our study was also compatible with those of another randomized controlled trial that suggested three to four passes [20]. Furthermore, there were no factors affecting the FNB cytopathological diagnosis rate in this study, which was also comparable with those of our previous study [21] that endoscopists who had achieved the learning curve of conventional EUS-FNA could overcome the difficulty of EUS tissue sampling, such as those due to chronic pancreatitis.

Regarding the learning curve, a previous study showed that more than 40 procedures could achieve a stable success rate of conventional EUS-FNA [21]. In this study, there was no obvious difference in the success rate of cytopathological diagnosis along the time sequence. Therefore, we determined that an endoscopist who was familiar with conventional EUS-FNA may perform CE-EUS-FNB without any difficulty.

As the limitations of this study, it was a retrospective study in a medical center. We did not enroll the diffident FNB needles, such as SharkCore and Procore needles. Also, there was no rapid on-site cytological evaluation.

In conclusion, CE-EUS-FNB required fewer needle passes for a correct diagnosis, but this did not provide additional benefit for the diagnostic yield improvement. For endoscopists who are proficient in conventional EUS-FNA, there will be no difficulty when performing CE-EUS-FNB.
